# Acupuncture Treatment Alleviating Pyroptosis on Asthma Inflammation in Mice via Micro‐RNA‐223/NLRP3 Pathway

**DOI:** 10.1155/mi/5665377

**Published:** 2026-07-12

**Authors:** Xia Wang, Li-Qi Yang, Yong Wang, Zhi-Hang Tang, Yue-Wen He, Wen-Qi Wu, Wu-Hua Ma

**Affiliations:** ^1^ Department of Anesthesia, The First Affiliated Hospital of Chongqing Medical University, Chongqing, China, cqmu.edu.cn; ^2^ Department of Integrated Traditional Chinese and Western Medicine, Fosun Chan Cheng Hospital China, Foshan, China; ^3^ Department of Anesthesia, The First Affiliated Hospital of Guangzhou University of Chinese Medicine, Guangzhou, China, gzhmc.edu.cn; ^4^ Department of Anesthesia, Sun Yat-Sen Memorial Hospital of Sun Yat-Sen University, Guangzhou, 510120, China, sysu.edu.cn

**Keywords:** acupuncture treatment, asthma, miR-223/NLRP3 inflammasome, pyroptosis, T cell immunity

## Abstract

**Background:**

Evidence has shown that acupuncture treatment could significantly reduce the activation inflammation in asthmatic mice. However, the underlying mechanism through which acupuncture influences asthma has not yet been elucidated.

**Methods:**

OVA‐induced asthma in NLRP3^‒/‒^ and wild‐type C57BL mice, and lipopolysaccharides (LPS) induced inflammation in 16HBE cell were established in vivo and in vitro. H&E, Masson, and Sirius staining, along with ELISA kits, Western Blot, PCR, lactate dehydrogenase (LDH), immunohistochemistry, and immunofluorescence techniques were employed to assess the miR‐223/NLRP3‐mediated pyroptosis pathway in mitigating asthma‐induced inflammation.

**Results:**

In vitro, acupuncture treatment alleviated airway inflammation in asthmatic mice, upregulated miR‐223 expression, inhibited the genes and proteins expression level in NLRP3 pathway, such as NLRP3, GSDMD, ASC, and Caspase‐1, it also lowered the presence of cytokines tied to T cells. Activation of the NLRP3 pathway seemed to make asthma symptoms worse, whereas acupuncture treatment effectively alleviated the inflammation by modulating the miR‐223/NLRP3 pathway. In vivo, after upregulating or downregulating miR‐223 expression in an LPS‐induced cell inflammation model, the expression levels of the NLRP3 pathway changed significantly. The dual‐luciferase assay revealed the specific interaction of miR‐223 to the NLRP3 gene, thereby suppressing its transcription.

**Conclusions:**

miR‐223‐3p can bind to NLRP3 and suppresses the expression of NLRP3 gene. Acupuncture treatment can activate miR‐223/NLRP3‐related pyroptosis pathway to regulate T‐lymphocyte immunity and effectively reduce airway inflammation in asthmatic mice.

## 1. Introduction

Asthma is an all‐too‐common chronic respiratory condition, impacting over 350 million individuals, ranging from kids to adolescents to adults, on a global scale [1]. It also imposes a huge economic burden on the global health care system [2]. This heterogeneous disease, commonly marked by persistent bronchial inflammation, is diagnosed based on a person’s history of breathing troubles, including hacking coughs, whistling sounds when breathing out, a sensation of tightness in the chest, and difficulty catching a full breath. The severity and frequency of these symptoms can shift like the tides, which vary over time and in intensity [3, 4]. Therefore, controlling inflammation in asthma to relieve symptoms and avoid future disease exacerbations is central to asthma treatment [3, 5]. Several studies have found that T lymphocytes, especially those with CD4^+^ markers, combined with abnormal cytokine secretion, may play an important role in causing asthma‐specific inflammatory responses [6].

The NLRP3 inflammasome is an intracellular hub built from three core components: NLRP3, ASC, and effector protein pro‐caspase‐1. When it encounters a specific stimulus, it will assemble to turn pro‐caspase‐1 into its active conformation. Once activated, caspase‐1 cleaves its substrates to pro‐IL‐1β, pro‐IL‐18, and gasdermin D (GSDMD), which will lead to inflammation and a special way of cell death [7]. This molecular pathway is very important in initiating the innate immune response. Numerous pieces of evidence have implicated abnormal activation responses of the NLRP3 inflammasome in hyperinflammation and cellular harm, shaping the disease course of chronic inflammatory diseases like asthma [8].

MicroRNAs (miRNAs), compact noncoding RNAs, typically consist of 22 nucleotides. Their primary role is to modulate gene expression following transcription, most of which is inhibition, but occasionally promote this process [9]. They are responsible for controlling about 30% of the human genome and managing gene networks that control normal cell activities and inflammatory reactions [10]. It is not surprising that emerging data further supports the association of miRNAs with airway inflammation in asthma [11]. MiR‐223 ranks as the most prevalent microRNA in peripheral blood platelets and may even function as a systemic homeostatic regulator [12]. The key role of miR‐223 has been consistently demonstrated in recent research studies. When bacteria invade, it can help cells respond and regulate our immune system [13]. This microRNA is also important in inflammation‐related diseases, as it can regulate transcription factors of several genes, such as C/EBPα, activation of NOD‐like receptors, ubiquitin ligases Roquin, E2F1, and the NF‐κB pathway [14]. Both sputum and peripheral blood reticular gene analyses of asthmatic patients suggest that miR‐223 is a negatively correlated factor regulating asthma inflammation. MiR‐223 affects the NLRP3 inflammasome‐mediated process in cells by targeting and binding to the 3 ’UTR of the NLRP3 gene [15]. As a practice originating from traditional Chinese medicine (TCM), acupuncture has treated diverse ailments with high safety and no side effects for several millennia [16]. Acupuncture has been shown to be beneficial for the treatment of various respiratory allergic inflammations, including asthma [17, 18]. Our previous data demonstrated that acupuncture effectively treated asthma by attenuating AHR, reducing mucin production, and alleviating pulmonary inflammatory responses [19]. However, detailed evidence clarifying the mechanistic actions of acupuncture against asthma remained sparse. Accumulating evidence from earlier studies indicates that acupuncture pretreatment can activate miR‐223, which represses the post‐transcriptional activity of the NLRP3 gene to inhibit the NLRP3/IL‐1β axis and then effectively reduce NLRP3 inflammasome‐mediated pyroptosis; this mechanism has therapeutic implications across various conditions but has not been mentioned in asthma treatment [18]. Therefore, our investigation aimed to create an ovalbumin (OVA)‐induced asthma model in mice, employing miR‐223 agomir and NLRP3‐knockout mice (NLRP3^‒/‒^) to delve deeper into the contribution of the NLRP3 inflammasome to asthma’s airway inflammation and to explore acupuncture’s role in managing these complications.

## 2. Materials and Methods

### 2.1. Animals

Female C57BL/6 mice—both wild‐type (WT) and NLRP3‐knockout strains, which were aged between 6 and 8 weeks—were purchased from Shanghai Medel Organisms Center, Inc. Before the experiments began, the rodents went through a week of acclimatization in a sterile environment under conditions of unhindered access to nourishment and hydration. The mice were raised under strictly controlled temperature conditions of 21 ± 2°C and a humidity level of 60% ± 10%, and they were subjected to a 12 h light–dark cycle within a cleanroom setting. Each group, consisting of six animals, was formed through a random assignment process prior to the experiments. In the event that an animal is lost or succumbs to any cause during the experiment, the relevant section is repeated to maintain the integrity of the data. The research adhered to institutional ethical standards, and all procedures involving animals received formal approval from the Animal Experimental Ethical Committee of Guangzhou University of Chinese Medicine (Approval Number TCMF1‐2021028, effective as of May 14, 2021).

### 2.2. Asthma Model and Acupuncture

C57BL/6, NLRP3^‒/‒,^ and normal mice were utilized to establish an asthma model through OVA exposure. Manual acupuncture was performed on treatment groups at the following acupoints: Dazhui (GV14), Feishu (BL13), and Zusanli (ST36). Sensitization was achieved with an OVA‐based protocol from days 0–14, involving 0.2 mL of saline infused with OVA (10 μg) plus 1 mg of aluminum hydroxide (Al(OH)_3_). Starting on day 15, mice in the acupuncture groups received treatment every 2 days for a total of seven sessions by the same acupuncturist, who was not involved in subsequent data analysis. Acupuncture was applied bilaterally at acupoints GV14, BL13, and ST36, as defined by the sixth edition of Experimental Acupuncture Science. After inserting sterile stainless‐steel needles (0.3 × 13 mm) to a depth of 3 mm, the acupoints were stimulated for 20 min, with 20 cycles of manipulation performed once every 5 min. From days 28–30, the mice were challenged daily for 20 min with 1% OVA aerosol. After the last challenge, anesthesia was induced with sodium pentobarbital for sample collection, euthanasia was performed via cervical dislocation, and the bodies were disposed of properly as per lab protocols after sample collection. The flow chart of the study is shown in Figure S1.

### 2.3. Serum and BALF Collection

1% pentobarbital sodium of 0.2 mL was used to complete anesthesia for mice; the eyeballs of mice were removed with forceps; blood samples were collected into an EP‐tube; and the supernatant was collected after standing at room temperature for 1–3 h and centrifuging the blood samples. Mice were fixed on the sampling table, and the long axes of the mouse body and limbs were kept in a tight state. The neck muscles were separated to fully expose the trachea, and a small indwelling needle was placed in the wall of the airway with an oblique opening into the trachea; then, the needle and trachea were fixed with a silk thread. Next, the abdomen was opened, the chest was exposed, and the left lung was ligated. Used a 1 mL sterile syringe to inject 0.5 mL of sterilized ice PBS into the right lung with a constant speed through the indwelling needle, slowly withdrawn, and then injected the fluid back again and all process were repeated 3 times, the liquid was BALF. Serum and BALF were placed in EP tubes and quickly stored in a refrigerator at −80°C after collection.

### 2.4. Histopathological Assessment

Histological staining was carried out on 4 µm paraffin sections from the left lung employing hematoxylin and eosin (H&E), Masson’s trichrome (Masson), and Sirius red (Sirius), which had been immediately collected and fixed in 4% paraformaldehyde. An optical microscope (BX51, OLYMPUS, Japan) was used to scan sections.

### 2.5. Cytokine Assessment

The concentrations of IgE, IL‐17, TGF‐β, and IL‐18 in serum, together with IL‐4, IL‐1β, and IFN‐γ in BALF samples, were measured by ELISA kits following manufacturer protocols (MEIMIAN, JiangSu, China).

### 2.6. Western Blot Analysis

Half of the right lung was transferred into RIPA buffer (Beyotime, Shanghai, China), lysed for 30 min, subjected to routine protein quantification, and then denatured by heating for 5 min. Thirty μg of the lysate was run on SDS‐PAGE, transferred, and incubated with milk for blocking. Immunoblotting was conducted with primary antibodies against GSDMD‐N (S10137), ASC (S67824), and β‐actin (S93473) (all from Cell Signaling Technology, CST, USA; 1:1000), NLRP3 (ab263899) (Abcam, Cambridge, UK; 1:1000), and caspase‐1/p20 (AF4005) (Affinity Biosciences, USA; 1:1000). These primary antibodies were paired with secondary antibodies conjugated to horseradish peroxidase (HRP; supplied by Abcam, ab6721, England), which were incubated overnight at a 1:5000 dilution. The resulting protein bands were then subjected to densitometric analysis via ImageJ software.

### 2.7. Real‐Time PCR

The MiniBest RNA Extraction Kit (Takara, Otsu, Japan) was used to purify total RNA, and a CFX Connect Detection System was employed for real‐time qPCR using iQ SYBR Green Supermix as the reaction master mix. β‐Actin served as the normalization reference. Table 1 lists the sequences of the specific primers.

**Table 1:** Sequences of the specific primers.

Gene	Forward	Reverse
miR‐223	GCGCGTGTCAGTTTGTCAAAT	AGTGCAGGGTCCGAGGTATT
U6	CTCGCTTCGGCAGCACA	AACGCTTCACGAATTTGCGT
GAPDH	CATCACTGCCACCCAGAAGACTG	ATGCCAGTGAGCTTCCCGTTCAG
NLRP3	ATTACCCGCCCGAGAAAGG	TCGCAGCAAAGATCCACACAG
ASC	CTTGTCAGGGGATGAACTCAAAA	GCCATACGACTCCAGATAGTAGC
Caspase‐1	AACAGAACAAAGAAGATGGCACA	CCAACCCTCGGAGAAAGAT
IL‐1β	GAAATGCCACCTTTTGACAGTG	TGGATGCTCTCATCAGGACAG
IL‐18	TGGAGACCTGGAATCAGACA	TGGGGTTCACTGGCACTT

### 2.8. Immunofluorescence

After dewaxing and rehydration, the samples were subjected to a 1 h blocking process with a 3% bovine serum albumin solution at ambient temperature. Post‐blocking, the sections were soaked in anti‐GSDMD antibody (CST, S12741) diluted 1:100 overnight at 4°C, followed by an additional hour of exposure to the matched secondary antibody (Abcam’s ab150074, at a ratio of 1:500) under room‐temperature conditions. The sections, after being washed multiple times in PBS, were ultimately mounted with a DAPI‐supplemented antifade reagent and then maintained at 4°C.

### 2.9. Immunohistochemistry

Sections, after dewaxing and rehydration, underwent a 1 h blocking incubation with 3% bovine serum albumin at room temperature. Once the blocking solution was taken off, the slides were left to incubate with ASC (S67824, CST) and NLRP3 (ab214185, Abcam Inc.) antibodies in a refrigerator for the night. The secondary antibody (ab205718, Abcam) was applied for a 20 min incubation period, subsequently followed by chromogen and counterstaining for visualization. The sections were then given a 10 min treatment with 0.1% hydrochloric acid, followed by a 5 min cleaning under running water. After dehydration through graded ethanol, the sections were sealed with neutral gum and then observed via light microscopy. Following dehydration using a gradient of ethanol, neutral gum was applied to mount the sections, which were then analyzed by a light microscope.

### 2.10. LDH Measurement in Serum

LDH in serum was detected using the CytoTox 96 kit (Nanjing JianCheng, Nanjing, China) by ELISA as instructed.

### 2.11. Cell Culture and Transfection

16HBE cells (HBE135‐E6E7, ATCCCRL‐2741(STR)) were seeded in a 6‐well tissue culture dish under 37°C conditions for a duration of 24 h. A transfection assay was performed to transfect cells with miR‐223 agomir and miR‐223 antagomir for 24 h using LipoRNAi (Beyotime, Shanghai, China). 6 h post exposure, the cell inflammation model was prepared by intervening in the cell Mod group with lipopolysaccharides (LPS), miR‐223 agomir, and miR‐223 antagomir.

### 2.12. Dual‐Luciferase Reporter Gene Detection

The StarBase database (http://starbase.sysu.edu.cn/) served to pinpoint potential binding sites, based on which WT and mutant‐type (MUT) vectors of TRPC1 (Promega, Madison, USA) were produced. Then, 293T cells were subjected to transfection with 0.5 μg of these vectors combined with either miR‐223‐5p mimic or NC using GP‐transfect‐Mate (GenePharma, Shanghai, China) as the transfection reagent. After 48 h, the luciferase activity was measured (Promega, Madison, USA).

## 3. Statistical Analysis

GraphPad Prism 5 (GraphPad Prism Software Inc., CA, USA) and SPSS 25.0 (SPSS Inc., USA) served as the statistical platforms. Normally distributed data were shown as mean ± SD ($\bar{x}\pm s$), whereas data not following a normal distribution were summarized as median and interquartile range (IQR). QQ plots were generated to assess normality, and variance homogeneity was evaluated via Levene’s test. For datasets with a normal distribution and homogeneity in variance, we employed one‐way ANOVA to compare groups, with the LSD method applied for follow‐up analyses. In cases of nonhomogeneous variance, we turned to the Welch test and the Dunnett T3 test for group‐wise comparisons. When normal distribution was not present, Kruskal–Wallis nonparametric tests were utilized for multigroup comparisons, with Bonferroni correction for post‐hoc testing. A *p*‐value below the conventional threshold of 0.05 was considered to denote statistical significance. No fewer than three replicates were completed for all experimental conditions.

## 4. Results

### 4.1. Effect of Acupuncture on the miR‐223/NLRP3‐Related Pyroptosis Pathway and T‐Cell Subsets in Asthma Inflammation in Mice

**Figure 1:**
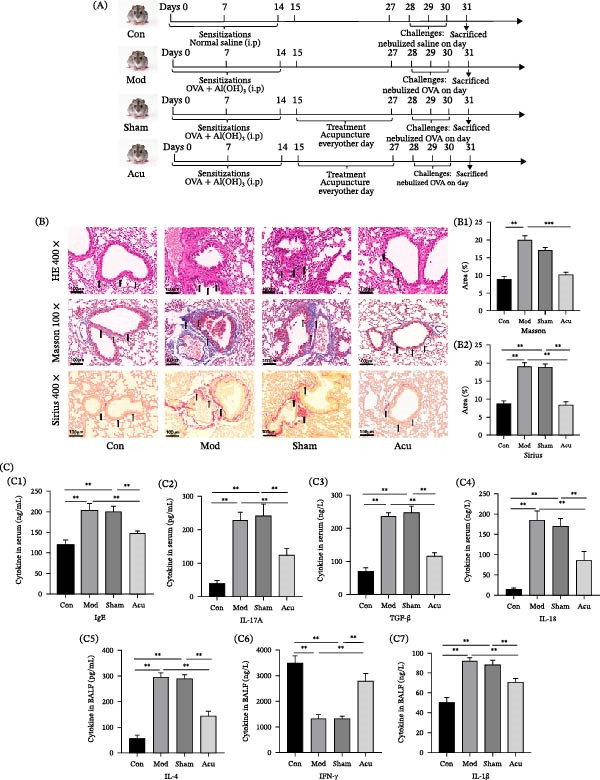
Effect of acupuncture on lung injury and cell factors in asthma inflammation in mice. Acupuncture treatment can effectively reduce airway inflammation, lung injury and T‐lymphocyte‐associated factors in Mod mice. Flow chart of groups of OVA‐induced asthma in mice (A); results of HE, Masson, and Sirius staining in four groups of mice (B), collagen deposition area histogram of Masson staining pear group (B1), collagen fiber area histogram of Sirius staining pear group (B2); IgE (C1), IL‐17A (C2), TGF‐β (C3), IL‐18 (C4) in serum samples and IL‐4 (C5), IFN‐γ (C6), and IL‐1β (C7) in BALF samples were assessed from each group. Data are mean ± SD, *n* = 6 per group.  ^∗^ means *p*  < 0.05;  ^∗∗^ means *p*  < 0.01;  ^∗∗∗^ means *p*  < 0.001.

To explore the impact of acupuncture treatment on asthmatic airway damage, we first established a murine model of OVA‐induced asthma (Mod). The model was then treated with either sham acupuncture (Sham) or acupuncture at targeted points (Acu) and the control group (Con). Each group included six mice; the sample for this section contained 24 mice in total (the modeling process is shown in Figure 1A).

1. Acupuncture intervention alleviated airway inflammatory responses and pulmonary tissue injury in an OVA‐induced asthma model.

H&E, Masson, and Sirius staining were applied to evaluate OVA‐induced lung injury. Microscopic examination revealed marked inflammatory cell infiltration within the trachea, along with bronchial mucosal edema and epithelial cell focal detachment in OVA‐challenged mice. In contrast, acupuncture therapy led to a notable reduction in inflammatory cell infiltration relative to the Mod and Sham groups (Figure 1B).

We examined the cytokine concentrations in BALF and serum samples. The Mod and Sham groups, when benchmarked against the control group, had exhibited a substantial serum level rise of IgE, IL‐17A, TGF‐β, and IL‐18. However, their expression levels were significantly reduced after acupuncture treatment (Figure 1C). Furthermore, the cytokine levels of IL‐4, IFN‐γ, and IL‐1β in BALF showed the same trend as those in the serum. Hence, the levels of T‐lymphocyte‐associated and NLRP3‐dependent pyroptotic inflammatory cytokines were substantially lowered post‐acupuncture treatment.

2. The effect of acupuncture treatment on the miR‐223/NLRP3‐mediated inflammasome pathway in asthmatic mice.

**Figure 2:**
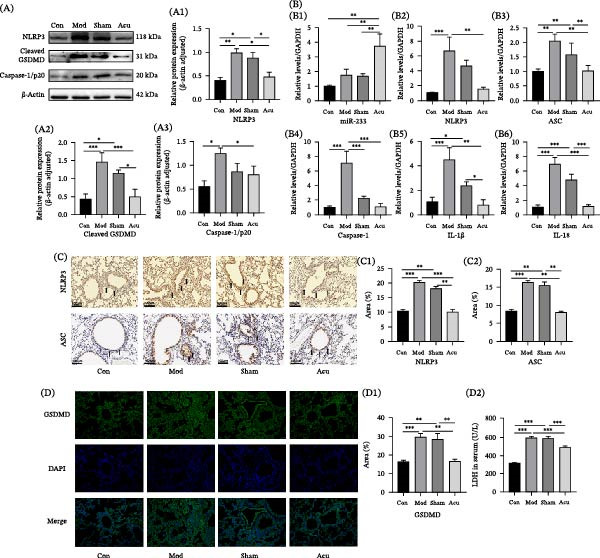
Impact of acupuncture on the miR‐223/NLRP3‐driven pyroptosis pathway in mice with asthma. Acupuncture treatment can effectively down‐regulate the miR‐223/NLRP3 pyroptosis signaling pathway in mice with asthma. The abundance of protein expression related to the NLRP3 pathway (A), including NLRP3 (A1), cleaved‐GSDMD (A2), and caspase‐1/P20 (A3). Expression levels of miR‐223/NLRP3‐related genes such as miR‐223 (B1), NLRP3 (B2), ASC (B3), Caspase‐1 (B4), IL‐1β (B5), and IL‐18 (B6) were examined by PCRs (B). Immunohistochemical expression of NLRP3 and ASC proteins in lung tissue (C) and the immunohistochemical area of NLRP3 (C1) and ASC (C2) proteins were calculated; immunofluorescence of GSDMD protein in lung tissue (D) and the immunofluorescence area of GSDMD (D1) and LDH content (D2) in serum were analyzed. Data are mean ± SD, *n* = 3 per group.  ^∗^ means *p*  < 0.05;  ^∗∗^ means *p*  < 0.01;  ^∗∗∗^ means *p*  < 0.001.

In our subsequent study, we delved into the gene and protein expressions of various factors in the miR‐223/NLRP3 inflammatory pathway. The Acu group displayed substantially lower expression levels of NLRP3, ASC, Caspase‐1, IL‐1β, and IL‐18 (Figure 2A,B) in comparison with the Mod group, while the miR‐223 gene was upregulated in the Acu group. Meanwhile, the changes in protein levels determined through Western blot analysis were consistent with the gene expression results. Specifically, protein levels rose in the Mod and Sham groups but dropped post‐acupuncture. Furthermore, immunohistochemical analysis was employed to pinpoint and quantify the NLRP3 and ASC levels in the pulmonary tissue of the animals. As depicted in Figure 2C, NLRP3 and ASC demonstrated clustering (seen as brown) in the Mod and Sham groups, whereas these expressions were markedly diminished in the Acu group, with no obvious aggregation around the bronchus.

3. The effect of acupuncture on NLRP3‐mediated pyroptosis in airway inflammation of asthmatic mice.

To identify the presence of GSDMD—a pivotal player in the NLRP3‐driven pyroptosis cascade—we utilized immunofluorescence techniques. As depicted in Figure 2D, the fluorescent expression of GSDMD in the Mod and Sham groups was markedly elevated relative to that in the Con group (green fluorescence) and was significantly reduced after acupuncture treatment. To further examine inflammation, we also measured LDH levels in serum, which showed similar changes observed in GSDMD protein expression.

In summary, the abundance of related proteins within the NLRP3‐mediated pyroptosis pathway was elevated as detected by WB. Data from immunohistochemistry and immunofluorescence, along with LDH levels, also confirmed that the NLRP3‐mediated pyroptosis pathway was activated in asthmatic mice. After acupuncture treatment, their expression levels in mice were significantly reduced, confirming that acupuncture could suppress the pyroptosis pathway involving NLRP3 in asthmatic mice.

### 4.2. Study of the Impact of miR‐223 Overexpression in Acupuncture‐Mediated Modulation of NLRP3‐Driven Pyroptosis and T‐Lymphocyte Subsets in Asthmatic Mice

Our previous study indicated that the expression levels of miR‐223 were upregulated following acupuncture treatment in OVA‐induced asthmatic mice. To further ascertain whether acupuncture treatment mitigates airway inflammation in asthmatic mice through activation of the miR‐223/NLRP3 axis, we used an miR‐223 agomir (GenePharma, Suzhou, China) to overexpress the miR‐223 gene in NLRP3‐knockout (NLRP3^‒^/^‒^) and WT mice. The impact of acupuncture on the miR‐223/NLRP3 pathway and T‐lymphocyte‐mediated immunity in asthmatic mice was then examined.

**Figure 3:**
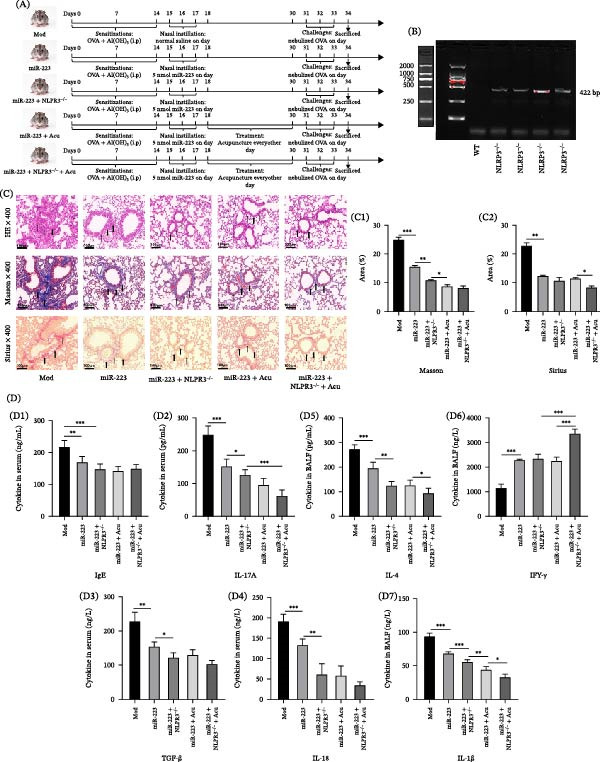
Effect of acupuncture on lung injury and cell factors in OVA‐sensitized asthmatic mice after miR‐223 overexpression. The impact of acupuncture on lung tissue damage and T‐cell immunity in OVA‐sensitized asthmatic mice after miR‐223 overexpression; a flow chart of groups after miR‐223 overexpression in mice (A); the genotyping results of NLRP3^‒/‒^ mice (B); results of HE, Masson, and Sirius staining in four groups of mice (C), per‐group histograms of collagen fiber area based on Masson staining (C1), and per‐group histograms of collagen fiber area based on Sirius staining (C2); IgE (D1), IL‐17A (D2), TGF‐β (D3), and IL‐18 (D4) in serum samples and IL‐4 (D5), IFN‐γ (D6), and IL‐1β (D7) in BALF samples were assessed from each group. Data are mean ± SD, *n* = 6 per group.  ^∗^ means *p*  < 0.05;  ^∗∗^ means *p*  < 0.01;  ^∗∗∗^ means *p*  < 0.001.

First, we identified and bred NLRP3^‒/‒^ mice (the genotyping results of NLRP3^‒/‒^ mice are shown in Figure 3B) and used a miR‐223 agomir to specifically induce high expression of miR‐223 in the mouse airways via nasal inhalation [20]. Twelve female NLRP3^‒/‒^ mice (5 to 6 weeks old) were randomly assigned to two groups using randomized allocation in a Microsoft Excel table: (1) the miR‐223 + NLRP3^‒/‒^ group, in which NLRP3^‒/‒^ mice overexpressed miR‐223, and (2) the miR‐223 + NLRP3^‒/‒^ + Acu group, in which NLRP3^-^/^-^ mice with miR‐223 overexpression received acupuncture treatment. Additionally, a cohort of 18 female C57BL/6J WT mice with an age range of 5–6 weeks received random distribution across three individual groups: (1) the Mod group (asthma model), (2) the miR‐223 group (miR‐223 overexpression), and (3) the miR‐223 + Acu group (miR‐223 overexpression with acupuncture treatment). Except for the Con group, all mice were induced with OVA to develop asthma, as described in Section 1. From day 15, mice in the miR‐223, miR‐223 + NLRP3^‒/‒^, miR‐223 + Acu, and miR‐223 + NLRP3^‒/‒^ + Acu groups received a daily nasal instillation of 5 nmol miR‐223 agomir for three consecutive days. Among them, the miR‐223 + Acu and miR‐223 + NLRP3^‒/‒^ + Acu groups received acupuncture at targeted points starting from day 18, following the same protocol as that of the Acu group in Section 1. Each group consisted of six mice, and the sample for this section contained 30 mice in total. (The modeling process is shown in Figure 3A).

1. The impact of acupuncture on lung tissue damage and T‐cell immunity in OVA‐sensitized asthmatic mice after miR‐223 overexpression.

H&E, Masson, and Sirius staining were used to evaluate the extent of OVA‐induced lung injury (Figure 3C). The micrographs revealed an abundance of inflammatory cells invading the trachea, and the bronchial mucosa of the mice showed congestion and edema, with some epithelial cells detaching in the Mod group. Upon miR‐223 overexpression, the severity of pulmonary pathological manifestations in all groups showed a marked abatement. In the miR‐223 + NLRP3^‒/‒^ and miR‐223 + Acu groups, lung inflammation was significantly reduced. These findings demonstrate that miR‐223 overexpression mitigates OVA‐induced airway inflammation in asthmatic mice. Moreover, lung improvement was more pronounced in mice that received acupuncture treatment or NLRP3 knockout compared to those that only overexpressed miR‐223. These results suggest that NLRP3‐knockout and acupuncture treatment may exert similar protective effects against airway inflammation in asthmatic mice. Furthermore, acupuncture may alleviate airway inflammation by activating the miR‐223 pathway and inhibiting NLRP3 signaling.

Next, the same cytokines—including IgE, IL‐17A, IL‐18, and TGF‐β in serum, as well as IFN‐γ, IL‐4, and IL‐1β in BALF—were quantified to assess inflammation levels (Figure 3D). Compared with the Mod group, cytokine levels associated with T‐lymphocyte activity and the NLRP3 pathway decreased following miR‐223 overexpression. Furthermore, these inflammatory factors showed an even greater downward trend in the NLRP3^‒/‒^ and Acu groups when contrasted with the miR‐223 overexpression group. This suggests that boosting miR‐223 levels mitigates airway inflammation in asthmatic mice and that NLRP3‐knockout or acupuncture treatment may further enhance and potentiate this effect.

2. The effect of acupuncture treatment on the NLRP3‐mediated pathway in asthmatic mice after miR‐223 overexpression

**Figure 4:**
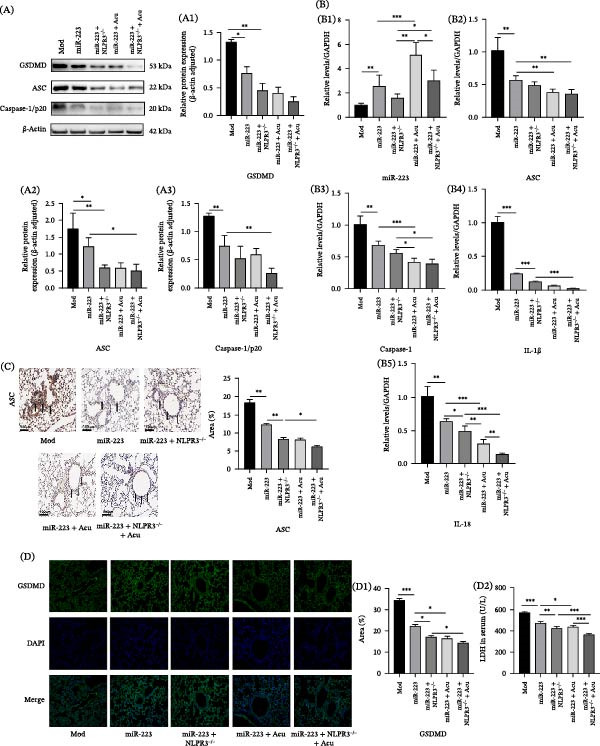
Effect of acupuncture treatment on the miR‐223/NLRP3‐mediated pyroptosis pathway in asthmatic mice after miR‐223 overexpression. Effect of acupuncture treatment on the NLRP3‐mediated pyroptosis pathway in asthmatic mice after miR‐223 overexpression. The levels of proteins tied to the NLRP3 inflammasome pathway (A), including NLRP3 (A1), GSDMD (A2), and caspase‐1/P20 (A3). Expression levels of miR‐223/NLRP3‐related genes such as miR‐223 (B1), ASC (B2), Caspase‐1 (B3), IL‐1β (B4), and IL‐18 (B5) were examined by PCRs (B). Immunohistochemical expression of ASC proteins in lung tissue (C), and the immunohistochemical area of ASC (C1) proteins were calculated; the immunofluorescence of GSDMD protein in lung tissue (D), the immunofluorescence area of GSDMD (D1), and LDH content (D2) in serum were analyzed. Data are mean ± SD, *n* = 3 per group.  ^∗^ means *p*  < 0.05;  ^∗∗^ means *p*  < 0.01;  ^∗∗∗^ means *p*  < 0.001.

We investigated the mRNA and protein levels within the miR‐223/NLRP3 pathway across various groups. Lower abundance of NLRP3, ASC, Caspase‐1, IL‐1β, and IL‐18 (Figure 4A,B) was observed following miR‐223 overexpression compared to the Mod group, while miR‐223 was upregulated in the corresponding groups. Moreover, a more pronounced decrease was detected in the NLRP3^‒/‒^ and Acu groups in contrast to the miR‐223 overexpression group. Additionally, protein profiles derived from Western blotting paralleled the observed changes in gene expression. Immunohistochemical staining was performed to localize and quantify ASC expression in lung tissues. As shown in Figure 4C, ASC aggregation (brown staining) was prominent in the Mod group, whereas its expression was obviously decreased in other groups, with no obvious aggregation around the bronchus.

3. Impact of acupuncture on NLRP3‐induced pyroptosis in mice with asthma after miR‐223 overexpression.

Immunofluorescence staining was applied for the identification of the GSDMD protein. As shown in Figure 4D, the immunofluorescence intensity of the GSDMD protein was highest in the Mod group (green fluorescence). A sharp decrease in GSDMD expression was found in the four treatment groups versus the Mod group. Among them, the miR‐223 + NLRP3^‒/‒^ + Acu group showed the lowest GSDMD expression in lung tissue. Similarly, LDH levels in the serum followed a comparable trend with GSDMD immunofluorescence. In relation to the Mod group, the amounts of LDH were significantly reduced in the miR‐223, miR‐223 + NLRP3^‒/‒^, miR‐223 + Acu, and miR‐223 + NLRP3^‒/‒^ + Acu groups, with statistically significant differences. Furthermore, compared with the miR‐223 group, LDH levels were significantly lower in the miR‐223 + NLRP3^‒/‒^, miR‐223 + Acu, and miR‐223 + NLRP3^‒/‒^ + Acu groups. Among these, the miR‑223 + NLRP3^‒/‒^ and miR‑223 + Acu groups exhibited markedly reduced LDH levels relative to the miR‑223^‒/‒^ + Acu group.

### 4.3. Effect of the Pyroptosis Gene NLRP3 on Acupuncture‐Mediated Regulation of miR‐223 and Pyroptosis‐Induced T‐Lymphocyte Immunity in Asthmatic Mice

Previous studies have implicated acupuncture treatment as protective within the miR‐223/NLRP3 axis. To further verify whether acupuncture inhibits the NLRP3 pathway by activating miR‐223 expression, we utilized NLRP3^‒/‒^ mice and NLRP3 agonist mice with nigericin sodium salt (Selleck, Houston, US) in this study. Additionally, a dual‐luciferase assay was performed in vitro using 293T cells to validate the targeted regulatory interaction between miR‐223 and the NLRP3 genes.

**Figure 5:**
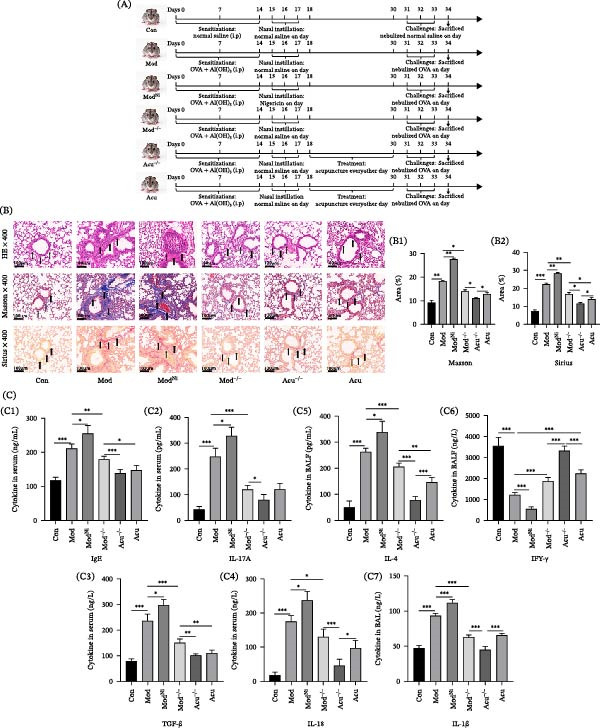
Effect of acupuncture on lung injury and cell factors after NLRP3 gene knockout and NLRP3 agonist treatment. Effect of acupuncture treatment on lung tissue injury and T‐cell immunity in asthmatic mice after NLRP3 gene knockout and NLRP3 agonist treatment. A flow chart of groups after NLRP3 gene knockout and NLRP3 agonist treatment in mice (A); results of HE, Masson, and Sirius staining in four groups of mice (B); a collagen deposition area histogram per group of mice subjected to Masson staining (B1), a collagen deposition area histogram per group of mice subjected to Sirius staining (B2); IgE (C1), IL‐17A (C2), TGF‐β (C3), and IL‐18 (C4) in serum samples; and IL‐4 (C5), IFN‐γ (C6), and IL‐1β (C7) in BALF samples were assessed from each group. Data are mean ± SD, *n* = 6 per group.  ^∗^ means *p*  < 0.05;  ^∗∗^ means *p*  < 0.01;  ^∗∗∗^ means *p*  < 0.001.

Twelve NLRP3^‒/‒^ female mice (aged 5–6 weeks) underwent randomization into two groups via a computer‐generated Excel table: the Mod^‒/‒^ group (NLRP3^‒/‒^ mice with standard modeling) and the Acu^‒/‒^ group (NLRP3^‒/‒^ mice with standard modeling and acupuncture treatment). Additionally, 24 female C57BL/6J mice received random distribution across four groups: the Con group, Mod group, Mod^Ni^ (NLRP3 agonist‐treated model) group, and Acu (acupuncture‐treated) group, using the same randomization method. Except for the Con group, all other five groups underwent intraperitoneal administration of 0.2 mL of OVA‐sensitizing solution three times on days 0, 7, and 14. The Con group received the same treatment, as described in Section 1. On day 15, mice in the Mod^Ni^ group received an injection of 0.4 mg/mL nigericin, with 4 mg/kg based on body weight, administered once daily for three consecutive days. The Acu^‒/‒^ and Acu groups received acupuncture treatment identical to that of the Acu group in Section 1. A total of 36 mice were included in this section (as shown in Figure 5A).

1. The impact of acupuncture on lung tissue injury and T‐cell immunity in asthma mice after NLRP3 gene knockout and NLRP3 activator treatment.

Significant inflammatory cell infiltration in the trachea was revealed by histological analysis employing H&E, Masson, and Sirius staining, accompanied by bronchial mucosal congestion and some shedding of epithelial cells in both the Mod and Mod^Ni^ groups. In contrast, NLRP3^‒/‒^ and Acu‐treated mice exhibited a significant reduction in inflammatory cell infiltration, with varying degrees of improvement. The overall lung histopathology in Acu^‒/‒^ mice was comparable to that of the Acu group (Figure 5B).

In order to quantify the effects of acupuncture treatment and the NLRP3 pathway on lung inflammation, cytokine levels were analyzed. In comparison with the Con group, the Mod and Mod^Ni^ groups saw a marked increase in IgE, IL‐17 A, TGF‐β, and IL‐18 serum levels. On the flip side, the Acu^‒/‒^, Mod^‒/‒^, and Acu groups saw a notable decline in these cytokine levels (Figure 5C). Similarly, the same pattern was observed for IL‐4, IFN‐γ, and IL‐1β in the serum. In contrast to the Acu^‒/‒^ group, the Mod^‒/‒^ and Acu groups exhibited a statistically significant difference in IL‐4, IFN‐γ, and IL‐1β levels. These results suggest that NLRP3 gene knockout and acupuncture treatment both contribute to a significant reduction in T‐lymphocyte‐associated and NLRP3‐mediated cytokine expression.

2. Effect of acupuncture treatment on the NLRP3 pathway of NLRP3^‒/‒^ and NLRP3‐agonist asthmatic mice.

**Figure 6:**
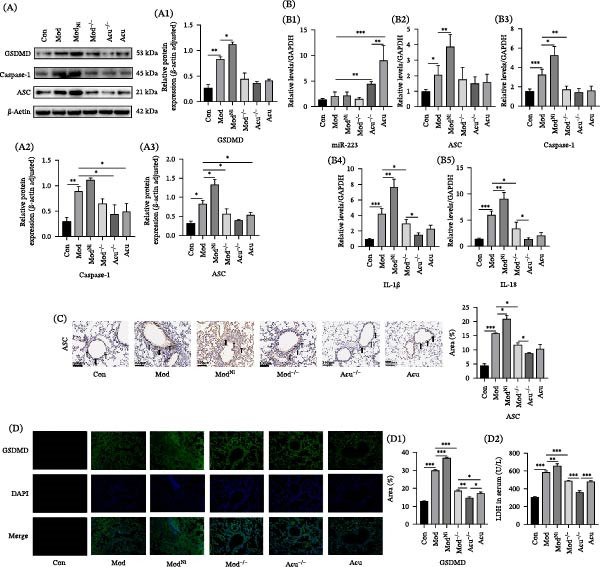
Influence of acupuncture treatment on the miR‐223/NLRP3‐mediated pyroptosis pathway after NLRP3 gene knockout and NLRP3 agonist treatment. Effect of acupuncture treatment on the NLRP3‐mediated pyroptosis pathway in NLRP3^‒/‒^ and NLRP3 agonist asthmatic mice. The levels of protein expression related to the NLRP3 pathway (A), including NLRP3 (A1), caspase‐1 (A2), and ASC (A3). Expression levels of miR‐223/NLRP3‐related genes such as miR‐223 (B1), ASC (B2), Caspase‐1 (B3), IL‐1β (B4), and IL‐18 (B5) were examined by PCRs (B). Immunohistochemical expression of ASC proteins in lung tissue (C), Immunohistochemical area of ASC proteins were calculated; Immunofluorescence of GSDMD protein in lung tissue(D), Immunofluorescence area of GSDMD (D1) and LDH content (D2) in serum was analyzed. Data are mean ± SD, *n* = 3 per group.  ^∗^ means *p*  < 0.05;  ^∗∗^ means *p*  < 0.01;  ^∗∗∗^ means *p*  < 0.001.

The abundance of both miR‐223 and key NLRP3 pathway components on both the transcriptional and translational levels was analyzed across different groups. As opposed to the control group, the manifestation of miR‐223 remained largely unchanged in the Mod, Mod^Ni^, and Mod^‒/‒^ groups. However, miR‐223 expression displayed a marked enhancement in the Acu and Acu^‒/‒^ groups over the Mod group. NLRP3 pathway genes at the transcriptional level (Figure 6A,B) differed significantly after NLRP3‐knockout and acupuncture treatment. These genes were markedly elevated in the Mod and Mod^Ni^ groups versus the Con group, with the Mod^Ni^ group exhibiting greater levels than those of the Mod group. The Acu^‒/‒^ group paralleled the Mod^‒/‒^ group in these gene expression. So, NLRP3 knockout alone does not significantly alter gene expression levels before or after acupuncture treatment. Similarly, protein levels of GSDMD, Caspase‐1, and ASC assessed by Western blotting were consistent with the gene expression results. The protein levels of these factors were elevated in the Mod and Mod^Ni^ groups, with the Mod^Ni^ group displaying the highest expression levels among all groups. However, their expression significantly decreased following NLRP3‐knockout or acupuncture treatment. To further assess ASC expression and localization, immunohistochemical staining was performed on lung tissues. As shown in Figure 6C, the Mod and Mod^Ni^ groups exhibited aggregation of ASC (brown staining) in lung tissue. In contrast, ASC expression exhibited markedly lower levels in the Mod^‒/‒^, Acu^‒/‒^, and Acu groups than in the Mod group, with no obvious aggregation around the bronchus. Compared with the Acu^‒/‒^ group, ASC expression was slightly higher in the Mod^‒/‒^ group (*p* < 0.05). However, the Acu^‒/‒^ and Acu groups exhibited similar levels of ASC protein expression.

Immunofluorescence detection of GSDMD protein expression (Figure 6D) revealed that the Mod and Mod^Ni^ groups had a notable rise in GSDMD fluorescence relative to the Con group (green fluorescence). In the Mod^‒/‒^, Acu^‒/‒^, and Acu groups, levels of GSDMD expression were notably lower than those in the Mod group. Conversely, both the Mod and Mod^Ni^ groups exhibited a substantial rise in serum LDH levels when pitted against the Con group. The Mod^Ni^ group experienced an even more pronounced increase in serum LDH levels than the Mod group did. On the flip side, the Mod^‒/‒^, Acu^‒/‒^, and Acu groups displayed a marked reduction in serum LDH levels over the Mod group. Additionally, compared to the Acu^‒/‒^ group, the Mod^‒/‒^ and Acu groups exhibited significantly higher serum LDH levels.

### 4.4. Changes in the NLRP3 Pathway and T‐Lymphocyte Inflammatory Factors After Upregulation or Downregulation of miR‐223 Expression In Vitro

The preceding three experiments have demonstrated that acupuncture can alleviate asthmatic inflammation by modulating the expression of miR‐223 to inhibit the NLRP3‐induced pathway and subsequently influence the release of T‐lymphocyte‐related inflammatory factors in vivo. However, it’s still unclear if miR‐223 has a direct impact on the NLRP3 pathway. We conducted an in vitro experiment using the human bronchial epithelial cell line of 16HBE in an LPS‐induced inflammatory model. By employing miR‐223 agomir (for miR‐223 overexpression) and miR‐223 antagomir (a miR‐223 inhibitor), we focused on the dynamic alterations of the NLRP3 pathway and T‐lymphocyte‐related inflammatory factors following the upregulation and downregulation of miR‐223 expression. Moreover, a dual‐luciferase reporter system was employed to confirm the selective binding linked miR‐223 to NLRP3.

1. The impact of altered miR‐223 expression on NLRP3 pathway activation and T‐cell‐derived inflammatory cytokines in 16HBE cells.

We measured the mRNA and protein expression levels of factors involved in the NLRP3 pathway, wherein the NLRP3, ASC, Caspase‐1, IL‐1β, and IL‐18 genes (Figure 7B) exhibited significantly downregulated gene expression in the miR‐223 agomir group as opposed to the Mod group. Meanwhile, the changes in protein levels detected by Western blot analysis were consistent with the gene expression results. Related protein levels were increased in both the Mod and miR‐223 antagomir groups and decreased after miR‐223 overexpression (Figure 7A). To assess the effects on T‐cell immunity in vitro, cytokine levels in BALF and serum were analyzed, too. Relative to the Con group, the Mod groups exhibited marked elevations in serum levels of IgE, IL‐17 A, TGF‐β, and IL‐18; however, there was a considerable decrease in cytokine concentrations within the miR‐223 agomir group (Figure 7C). Similarly, the cytokines IL‐4, IFN‐γ, and IL‐1β in BALF followed the same trend as those in the serum. No statistically significant differences emerged when comparing the Mod group with the miR‐223 antagomir group regarding the levels of IgE, IL‐17 A, TGF‐β, IL‐18, IL‐4, IFN‐γ, and IL‐1β. These results suggest that after overexpressing the miR‐223 gene, the T‐lymphocyte inflammatory response was markedly alleviated.

Figure 7Changes in the NLRP3 pathway and T‐lymphocyte inflammatory factors after upregulation or downregulation of miR‐223 expression in vitro. The levels of protein expression related to the NLRP3 pathway (A), including NLRP3 (A1), GSDMD (A‐2), ASC (A3) and caspase‐1/p20 (A4). Expression levels of NLRP3‐related genes such as NLRP3 (B1), ASC (B2), Caspase‐1 (B3), IL‐1β (B4), IL‐18 (B5) were examined by PCRs (B). IgE (C1), IL‐17A (C2), TGF‐β (C3), and IL‐18 (C4) in serum samples and IL‐4 (C5), IFN‐γ (C6), and IL‐1β (C7) in BALF samples were assessed from each group. Data are mean ± SD.  ^∗^ means *p*  < 0.05;  ^∗∗^ means *p*  < 0.01;  ^∗∗∗^ means *p*  < 0.001.

**Figure:**
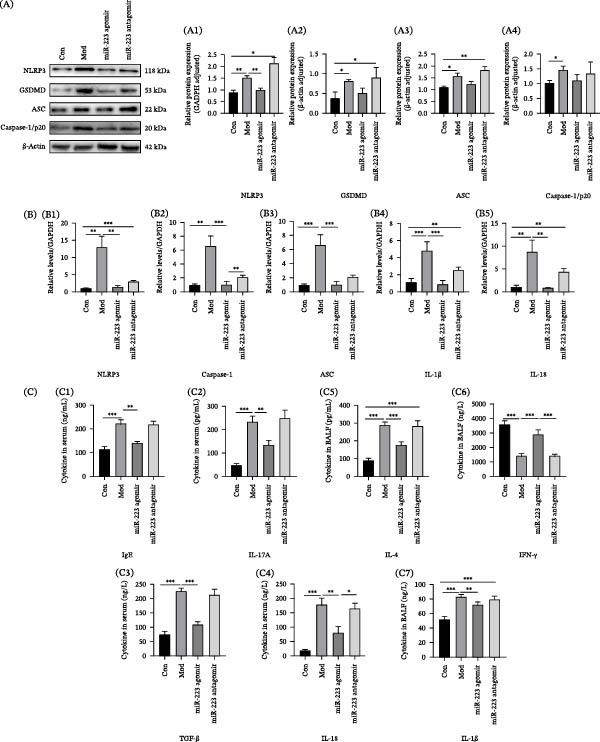

**Figure:**
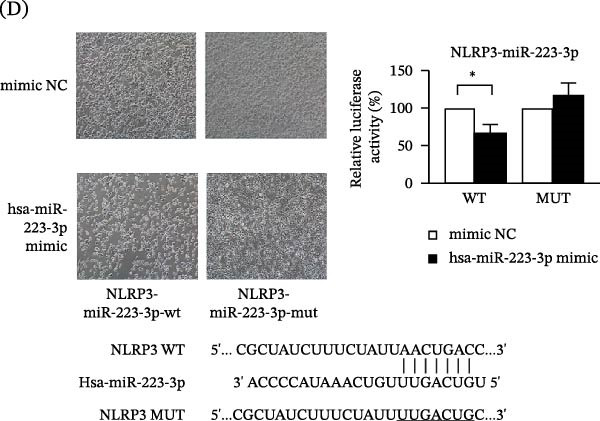

2. The dual luciferase assay evidenced the targeted association of miR‐223 with NLRP3.

To determine whether NLRP3 and miR‐223 interact, a bioinformatics study was conducted to identify possible binding locales between the NLRP3 3′ UTR and miR‐223. Complementarity between the miR‐223 sequence and the NLRP3 3′‐UTR was validated. Subsequent reporter assays using the dual‐luciferase system confirmed that boosting miR‐223 expression reduced luciferase activity driven by the WT NLRP3 3′‐UTR, whereas this effect was lost with the mutant construct (*p* < 0.05). In contrast, mimic NC exhibited no discernible variation in luciferase levels between the WT and the 3′‐UTR mutant. Together, these results demonstrate that NLRP3 is directly targeted by miR‐223 (Figure 7D).

## 5. Discussion

**Figure 8:**
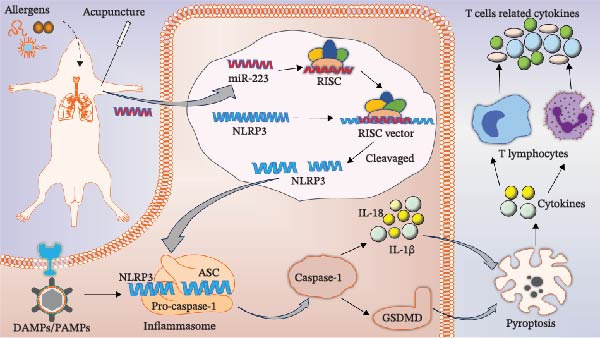
Mechanistic map of acupuncture treatment regulates the miR‐223/NLRP3‐mediated pyroptosis pathway, thereby influencing the release of T‐lymphocyte‐related factors and reducing airway inflammation in asthma.

Asthma is a chronic airway disease triggered by environmental exposure, characterized by telltale signs like whistling sounds when you breathe out, difficulty breathing, a constricted chest, persistent coughing, and limited airflow, which can develop rapidly and sometimes resolve spontaneously [21]. The present study focused on the function of the miR‐223/NLRP3 pathways in asthmatic mice and the therapeutic effects of acupuncture treatment through experiments. Following acupuncture treatment, results demonstrated that there was an increase in the miR‐223 gene’s presence in murine lungs, a suppression of the NLRP3 pathway, and a regulation of T‐lymphocyte subsets, thereby alleviating airway inflammation. Moreover, miR‐223 agomir was used to stabilize the enhanced levels of miR‐223 in the lungs of both WT and NLRP3‐knockout asthma subjects, aiming to verify whether acupuncture treatment inhibits the NLRP3 pathway by activating miR‐223. Finally, an OVA‐induced animal model was established using NLRP3^‒/‒^ mice and NLRP3 agonist nigericin. A reverse validation experiment showed that acupuncture treatment increased miR‐223 expression, while activation or inhibition of the NLRP3 pathway had little effect on miR‐223 gene expression levels. Ultimately, these findings confirmed that acupuncture treatment alleviates airway inflammation in asthmatic mice by activating miR‐223, which in turn inhibits the NLRP3‐mediated pyroptosis pathway and regulates immune balance in T lymphocytes, playing a protective role in asthma (the mechanical map is shown in Figure 8).

The establishment of an asthma model using an OVA as an allergen and aluminum hydroxide as an adjuvant (OVA‐AAI) injection has become a classic method widely used in pathophysiological studies related to bronchial asthma [22, 23]. The hallmark of this model is a significant spike in IgE levels within the blood, accompanied by airway inflammation, an increase in epithelial thickness, and a proliferation of mucus‐producing cells—symptoms that mirror the telltale signs of asthma in humans [24]. Following this modeling approach, we successfully established an asthma model, as confirmed by the pathological results from HE, Masson, and Sirius staining, along with IgE level measurements in the serum. In this study, the acupoints Dazhui (GV14), Feishu (BL13), and Zusanli (ST36) were selected for acupuncture treatment in asthmatic mice. Dazhui (GV14) belongs to the Du meridian, known as the “Confluence of All Yang,” and has the function of dispersing Yang, promoting sweating, and relieving asthma. Feishu (BL13), a key point on the Foot‐Taiyang Bladder Meridian, can regulate lung qi and relieve cough. Zusanli (ST36), a key point along the Stomach Meridian, is known for its role in regulating the functions of the spleen and lungs, tonifying blood and qi, bolstering the immune system, and maintaining overall qi balance. Therefore, Zusanli was chosen as a supporting acupoint. Several studies have used Dazhui and Feishu as acupoints, revealing the efficacy of acupuncture in modulating mucosal and cellular immunity in individuals suffering from allergic asthma [25]. In 2021, a Harvard research team confirmed that low‐intensity electroacupuncture stimulation at Zusanli markedly diminishes the presence of proinflammatory cytokines in mice with inflammation. The observed effect was attributed to the activation of the vagus nerve‐adrenal axis, mediated by the PROKR2 nerve [26].

MiR‐223 is pivotal in the innate immune response, particularly in relation to asthma [27]. Research has revealed that miR‐223‐3p suppresses NLRP3 gene activation through direct interaction with its 3′ UTR, thereby reducing the expression of inflammatory cytokines and chemokines, leading to attenuated airway inflammation [28, 29]. Acupuncture treatment has been found to activate the miR‐223/NLRP3 pathway, contributing to anti‐inflammatory effects in various diseases [30]. However, the specific mechanism by which acupuncture activates the miR‐223/NLRP3‐driven pyroptosis pathway in asthma has not yet been explored. This study investigated the changes in the miR‐223/NLRP3 pathway in asthmatic mice following acupuncture treatment. The findings indicated that, when compared with untreated mice, asthmatic rodents experienced a substantial boost in miR‐223 expression within their lung tissue following acupuncture. Meanwhile, key genes of the NLRP3 pathway dropped, together with IL‐1β and IL‐18. Further WB analysis unveiled that protein levels in the NLRP3 inflammasome pathway exhibited a similar decreasing trend after acupuncture treatment. Additionally, IL‐1β levels in the BALF and IL‐18 levels in the serum followed the same trend observed in the WB and PCR results, indicating that acupuncture treatment effectively reduced inflammatory factors in the BALF and serum of asthmatic mice. These findings confirmed the regulatory effect of acupuncture treatment on the miR‐223/NLRP3‐mediated pyroptosis pathway in asthmatic mice. In 2021, researchers demonstrated that acupuncture drives the vagal–adrenal axis through PROKR2‐mediated nerve fibers, thereby alleviating lipopolysaccharide (LPS)‐induced mouse inflammation [26]. However, whether acupuncture upregulates miR‐223 via specific subtypes of nerve fibers remains to be further investigated.

NLRP3 is associated with numerous inflammatory diseases in humans [31]. Studies using OVA‐induced asthma models in NLRP3^‒/‒^, IL‐1β^‒/‒^, ASC^‒/‒^, and Caspase‐1^‒/‒^ mice found that NLRP3 inflammasome pathway knockout significantly reduced airway inflammation, suggesting that NLRP3 inflammasome activation contributes to allergic asthma [32, 33]. Furthermore, as a transcriptional regulator of NLRP3, it also induces Th2 cell differentiation and promotes asthma symptoms through a noninflammasome‐dependent mechanism [34]. However, the specific mechanism of the NLRP3 inflammasome in asthmatic airway inflammation requires more in‐depth exploration. In this study, NLRP3^‒/‒^ mice and NLRP3 agonist‐treated mice were used to create an OVA‐induced asthma condition. Pathological analysis using H&E and Masson staining of lung tissue, along with IgE level measurements in the serum, revealed that the Mod^Ni^ group exhibited more severe lung structural damage than the Mod group. In contrast, the NLRP3^‒/‒^ mice exhibited a marked decrease in inflammatory cells and mucus production within the bronchial lumen. Furthermore, after acupuncture treatment, the lung structure in the Acu group resembled that of the NLRP3^‒/‒^ group, with minimal inflammatory cell infiltration and mucus secretion. Similarly, IgE levels in all groups followed a comparable trend.

In the current investigation, PCR, WB, and ELISA techniques were used to determine the gene expression, protein abundance, and cytokine levels associated with the miR‐223/NLRP3 inflammasome pathway. After acupuncture treatment, the quantity of miR‐223 shot up in both normal and NLRP3^‒/‒^ mice, but nothing much changed in the other groups. Additionally, the Mod^Ni^ group manifested a considerable rise in the activity of genes like ASC and IL‐18, coupled with heightened levels of proteins and a boost of cytokines in both serum and BALF, underscoring the NLRP3 inflammasome being overactivated. In the NLRP3^‒/‒^ cohort, a notable decrease in these physiological markers was observed when contrasted with the Mod group. Post‐acupuncture therapy, the levels of components within the NLRP3 inflammatory pathway saw a general decline across the board in every group. A direct association exists between NLRP3 inflammasome activation and asthmatic airway inflammation, as supported by these data, and inhibiting the NLRP3 inflammasome pathway can slow the inflammatory progression of asthma. Furthermore, acupuncture treatment effectively inhibits the NLRP3 inflammasome pathway, thereby reducing airway inflammation in asthma. T‐lymphocyte‐related immunity, including the balance of Th1/Th2 and Treg/Th17 cells, is key to the onset and advancement of asthma. Studies indicate that employing an NLRP3 inflammasome inhibitor can dramatically cut down on airway hypersensitivity and inflammation, and it also tends to redress the equilibrium among these immune cell subsets in mice with asthma [35].

Other studies have confirmed that inhibiting the NLRP3 inflammasome pathway can reestablish Th1/Th2 homeostasis in OVA‐induced asthmatic mice, thereby achieving a therapeutic effect against asthma [35, 36]. In our study, we noted a marked uptick in cytokines in the agonist group, contrasting with the Mod group, which saw a substantial downturn in IFN‐γ levels. This trend aligns with the typical characteristics of bronchial asthma, where proinflammatory factors (like TGF‐β, IL‐4, and IL‐17 A) increase while anti‐inflammatory factors (IFN‐γ) decrease. The NLRP3^‒/‒^ group showcased notably lower levels of inflammation when contrasted with the Mod group, and these changes were consistent across different samples. The data suggests that the NLRP3 inflammasome pathway acts as a critical mediator in regulating T‐lymphocyte immunity in asthma‐related inflammation. After acupuncture treatment, T‐lymphocyte cytokines exhibited similar changes across all groups, suggesting that acupuncture can regulate the release of T‐lymphocyte‐related factors by inhibiting the NLRP3‐mediated pyroptosis pathway, thereby reducing airway inflammation.

Additionally, previous studies have demonstrated that miR‐223 directly governs NLRP3 gene expression through interaction with its 3′ UTR binding site [37]. In our study, the dual‐luciferase assays confirmed that miR‐223 inhibits the NLRP3 gene, thereby suppressing the NLRP3‐mediated pyroptosis pathway. It is worth noting that our findings do not explicitly define the immune‐mediating molecular pathways connected to acupuncture treatment for asthma. Considering the intricate relationships among immune system cells, the molecular mechanisms linking acupuncture and NLRP3 pyroptosis require further in vitro studies for validation. Furthermore, in this study, we used mice with a complete NLRP3 gene knockout. While our results confirm that NLRP3 gene knockout alleviates pulmonary inflammation in asthma, it remains unclear whether systemic knockout of this gene may lead to pathological changes in other organs or tissues, potentially influencing pulmonary symptoms in asthmatic mice. Lastly, clinical sample collection was not included in this study. Acupuncture’s therapeutic outcomes at GV14, BL13, and ST36 in treating asthma will be a key focus in future research. Several limitations exist in our study. First, miR‐223 antagonist injections were not administered during acupuncture treatment in mice to determine whether this intervention reverses the inhibitory effects of acupuncture on the NLRP3 pathway and inflammation. Additionally, the global NLRP3‐knockout mice confirmed that knockout of the NLRP3 gene ameliorated pulmonary inflammation in asthma, but whether global deletion of this gene induced pathological changes in other organs or tissues, thereby influencing pulmonary symptoms in asthmatic mice, remains to be further explored.

## 6. Conclusion

To the best of our knowledge, no prior study has investigated the process through which acupuncture modulates the miR‐223/NLRP3‐induced pyroptosis pathway, thereby influencing the release of T‐lymphocyte‐related factors and reducing airway inflammation in asthma. The research offers fresh insights into how acupuncture can tackle airway inflammation in asthma, laying down a solid theoretical foundation. Moreover, this study is the first to delve into how boosting miR‐223 and suppressing NLRP3 in mice impacts their airways, demonstrating how acupuncture alleviates airway inflammatory responses in an asthma model. This was achieved via regulation of the miR‐223/NLRP3‐mediated pyroptosis pathway and modulating T‐lymphocyte subsets. The study employed multiple levels of validation, including both positive and negative controls, integrating in vitro and in vivo experimental approaches to comprehensively confirm the effects of acupuncture.

**Nomenclature**
NLRP3: NOD‐like receptor protein 3
ASC: Apoptosis‐associated speck‐like protein
PRR: Pattern recognition receptor
BALF: Bronchoalveolar lavage fluid
IgE: Immunoglobulin E
WT: Wild type
MUT: Mutant type
TGF‐β: Transforming growth factor‐beta
IFN‐γ: Interferon‐γ
GSDMD: Gasdermin D
miR‐223: MicroRNA‐223
C/EBPα: CCAAT/enhancer‐binding protein alpha
E2F1: E2F transcription factor 1
NF‐κB: Nuclear factor kappa‐B
TCM: Traditional Chinese medicine
OVA: Ovalbumin
NLRP3^‒/‒^: NLRP3 gene knockout
HE: Hematoxylin and eosin staining
Masson: Masson’s trichrome staining
Sirius: Sirius red staining
PROKR2: Prokineticin receptor 2.

## Author Contributions

Experimental work (design and performance) was conducted by Xia Wang, Li‐Qi Yang, Yong Wang, Zhi‐Hang Tang, Yue‐Wen He, Wen‐Qi Wu, and Wu‐Hua Ma. Writing of the original draft was done by Xia Wang, Li‐Qi Yang, Yong Wang, and Wu‐Hua Ma. Data interpretation and statistical analysis were handled by Zhi‐Hang Tang, Yue‐Wen He, and Wen‐Qi Wu. Critical revision of the manuscript was performed by Wu‐Hua Ma.

## Acknowledgments

ChatGPT and Deepseek were used to assist with the translation and language polishing of this manuscript.

## Funding

Funding was provided by the National Natural Science Foundation of China (Grant 82074357).

## Disclosure

All authors provided input in the conception and design. Having reviewed prior versions of the manuscript, all authors approved the final version.

## Ethics Statement

Approval for the animal ethics application was given by the Animal Experimental Ethics Committee of the First Affiliated Hospital of Guangzhou University of Chinese Medicine (Approval Number TCMF1‐2021028). The research followed the ethical standards laid out in (1) the 1964 Declaration of Helsinki and its later amendments; (2) the U.K. Animals (Scientific Procedures) Act 1986 and associated guidelines; (3) EU Directive 2010/63/EU for animal experiments; and (4) the National Research Council’s Guide for the Care and Use of Laboratory Animals.

## Consent

Informed consent was obtained from all individual participants.

## Conflicts of Interest

The authors declare no conflicts of interest.

## Supporting Information

Additional supporting information can be found online in the Supporting Information section.

## Supporting information


**Supporting Information** Additional file 1: Figure S1: The flow chart of study. Figure S2: Supporting file for original images.

## Data Availability

The evidence of this study may be requested via the corresponding author. Public access to these data is restricted owing to privacy or ethical considerations.
