# Supplementary material for: Acupuncture Treatment Alleviating Pyroptosis on Asthma Inflammation in Mice via Micro‐RNA‐223/NLRP3 Pathway
Source: Mediators Inflamm. 2026 Jul 12;2026:5665377. doi: 10.1155/mi/5665377 (PMC13358362; doi:10.1155/mi/5665377)
Supplement: Supplementary file 1 — Supporting Information Additional file 1: Figure S1: The flow chart of study. Figure S2: Supporting file for original images. [file MI-2026-5665377-s001.zip › 5665377.f1/Fig.S1 supplement file for flow chart.pdf]

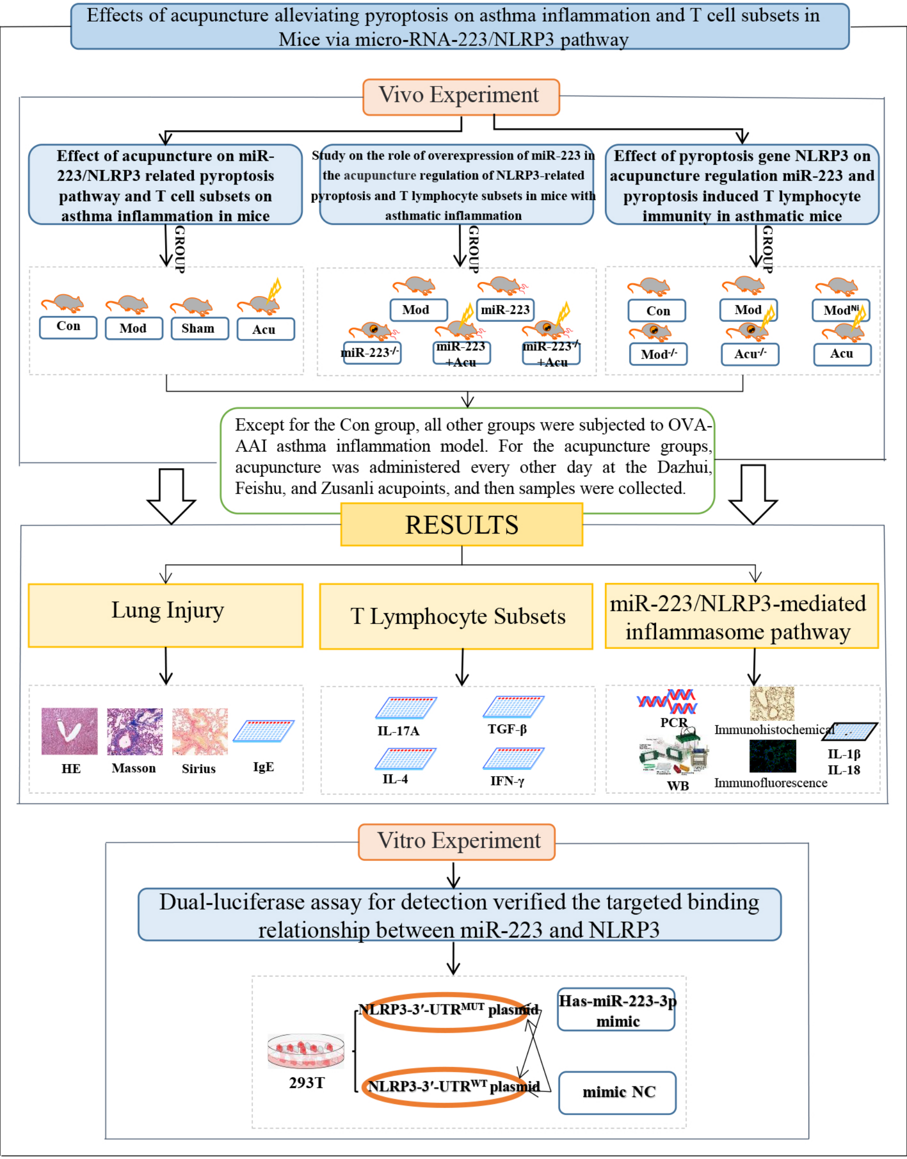

Effects of acupuncture alleviating pyroptosis on asthma inflammation and T cell subsets in Mice via micro-RNA-223/NLRP3 pathway
Vivo Experiment
Effect of acupuncture on miR-223/NLRP3 related pyroptosis pathway and T cell subsets on asthma inflammation in mice
Study on the role of overexpression of miR-223 in the acupuncture regulation of NLRP3-related pyroptosis and T lymphocyte subsets in mice with asthmatic inflammation
Effect of pyroptosis gene NLRP3 on acupuncture regulation miR-223 and pyroptosis induced T lymphocyte immunity in asthmatic mice
GROUP
GROUP
GROUP
Con
Mod
Sham
Acu
Mod
miR-223
miR-223-/-
miR-223 +Acu
miR-223-/- +Acu
Con
Mod
ModNi
Mod-/-
Acu-/-
Acu
Except for the Con group, all other groups were subjected to OVA-AAI asthma inflammation model. For the acupuncture groups, acupuncture was administered every other day at the Dazhui, Feishu, and Zusanli acupoints, and then samples were collected.
RESULTS
Lung Injury
T Lymphocyte Subsets
miR-223/NLRP3-mediated inflammasome pathway
HE
Masson
Sirius
IgE
IL-17A
TGF-β
IL-4
IFN-γ
PCR
Immunohistochemical
WB
Immunofluorescence
IL-1β
IL-18
Vitro Experiment
Dual-luciferase assay for detection verified the targeted binding relationship between miR-223 and NLRP3
293T
NLRP3-3′-UTRMUT plasmid
NLRP3-3′-UTRWT plasmid
Has-miR-223-3p mimic
mimic NC
