# Supplementary material for: Acupuncture Treatment Alleviating Pyroptosis on Asthma Inflammation in Mice via Micro‐RNA‐223/NLRP3 Pathway
Source: Mediators Inflamm. 2026 Jul 12;2026:5665377. doi: 10.1155/mi/5665377 (PMC13358362; doi:10.1155/mi/5665377)
Supplement: Supplementary file 1 — Supporting Information Additional file 1: Figure S1: The flow chart of study. Figure S2: Supporting file for original images. [file MI-2026-5665377-s001.zip › 5665377.f1/Fig.S2 supplementary file for original images.docx]

Dear Editor,

We are pleased to submit our manuscript titled **"Acupuncture treatment alleviating pyroptosis on asthma inflammation in Mice via micro-RNA-223 /NLRP3 pathway"**

In this study, the Western blot (WB) data were generated in three independent sections, and I have retained the original images for each part. However, for the first and second parts, due to my inexperience at the time, the full membranes were cropped during processing. That said, if you examine the cropped sections of NLRP3, GSDMD, and Caspase-1 from Section 1 and 2, you will notice that they were originally derived from the same intact image(s). The molecular weight marker positions can also be cross-referenced with the full membrane structure in Part 3 for verification (at least three original Western blot (WB) images for each protein in our study were supplied).

We hope this clarification demonstrates that all WB data in this study were faithfully obtained through our rigorous experimental work, with no manipulation or misrepresentation involved. Please let us know if further information is required.

Best regards,

The raw WB images of the three proteins in Fig. 2A

NLRP3（110kda）:

120kda

120kda

cleaved-GSDMD (31kda):

25 kda

25 kda

Caspase-1/P20 (20kda):

10 kda

10 kda

The raw WB images of the three proteins in Fig. 4A

GSDMD (53kda):

45kda

45kda

45kda

ASC (22kda):

30kda

30kda

30kda

Caspase-1/p20 (20kda):

10kda

10kda

10kda

The raw WB images of the three proteins in Fig. 6A

GSDMD (53kda):

45kda

45kda

45kda

Caspase-1 (45kda):

45kda

45kda

45kda

ASC (22kda):

30kda

30kda
